# Supplementary material for: Using cancer profiles to identify synthetic lethal therapeutic targets and predictive biomarkers in cancer gene dependency data
Source: Bioinformatics. 2026 May 27;42(6):btag337. doi: 10.1093/bioinformatics/btag337 (PMC13281939; doi:10.1093/bioinformatics/btag337)
Supplement: btag337_Supplementary_Data [file btag337_supplementary_data.zip › SupplementaryData.docx]

**Supplementary Data**

***Data Files***

DepMine uses a number of datafiles which are either supplied with the code or can be downloaded from a range of different sources :

| **File** | **Data** | **Web Page** | **Version** |
| --- | --- | --- | --- |
| CRISPRGeneDependency.csv | gene dependency | https://depmap.org/portal/download/all/ | Public 23Q2 |
| Model.csv | cell line information | https://depmap.org/portal/download/all/ | Public 23Q2 |
| OmicsSomaticMutations.csv | mutations | https://depmap.org/portal/download/all/ | Public 23Q2 |
| OmicsCNGene.csv | copy number | https://depmap.org/portal/download/all/ | Public 23Q2 |
| OmicsFusionFiltered.csv | gene fusions | https://depmap.org/portal/download/all/ | Public 23Q2 |
| OmicsExpressionProteinCodingGenesTPMLogp1.csv | expression | https://depmap.org/portal/download/all/ | Public 23Q2 |
| c1.all.v2023.1.Hs.json.txt | chromosomes | https://data.broadinstitute.org/gsea-msigdb/msigdb/release/2023.1.Hs/ | 2023.1.Hs |
| hgnc_complete_set.json | HUGO gene names | https://www.genenames.org/download/archive/ | Current |
| c2.cp.reactome.v2023.2.Hs.symbols.gmt | pathways | https://data.broadinstitute.org/gsea-msigdb/msigdb/release/2023.2.Hs/ | 2023.2.Hs |
| Homo_sapiens.GRCh38.110.refseq.tsv | ENSEMBLE reference sequences | https://www.ensembl.org/Homo_sapiens/Info/Index | GRCh38 |
| ChromosomeLengths.csv | Chromosome lengths | local |  |
| ExpressionThresholds.csv | Expression thresholds | local |  |
| Assignment_Solution_Activities.tsv | Mutation signature assignments | local |  |
| Final_GOF_all_table | Annotated GoFs | local |  |
| AB_Dep_Profiles | cancer profiles | local |  |

**Supplementary Table 1** - Table of cancer profile definitions referenced in the main text.

| Profile Name | Flat Definition* | Cell lines matched |
| --- | --- | --- |
| **PTEN_LOSS** | **-pten \| !pten** | 179 |
| **PTEN_LOSS_UTER** | **(-pten \| !pten) & %uter** | 22 |
| **BRAF_ACT_SKIN** | **/braf+ & %skin** | 75 |
| **MAPK_ACTIVATED** | **/hras:g12?,g13?,q61? \| /kras:g12?,g13?,q61? \| /nras:g12?,g13?,q61? \| /raf1:s257l,s259f \| /braf+** | 451 |
| **MYC_UP** | **+myc \| >myc** | 340 |
| **CCND3_OUTLIERS** | **<1ccnd3 \| >1ccnd3** | 379 |
| **CCND3_CENTRAL** | **# ^ (<1ccnd3 \| >1ccnd3)** | 1485 |
| **ARID1A_LOSS** | **!arid1a \| -arid1a** | 135 |
| **ARID1B_LOSS** | **!arid1b \| -arid1b** | 49 |
| **SMARCA2_LOSS** | **!smarca2 \| -smarca2** | 157 |
| **SMARCA4_LOSS** | **!smarca4 \| -smarca4** | 76 |
| **EP300_LOSS** | **!ep300 \| -ep300** | 76 |
| **CREBBP_LOSS** | **!crebbp \| /crebbp-** | 103 |
| **APC_LOSS** | **!apc \| -apc** | 100 |
| **RB1_LOSS** | **!rb1 \| -rb1** | 95 |
| **SMAD2_LOSS** | **!smad2 \| -smad2** | 78 |
| **SMAD4_LOSS** | **!smad4 \| -smad4** | 159 |
| **DCC_LOSS** | **!dcc \| -dcc** | 112 |
| **18Q21_DEL** | **-18q21** | 129 |
| **18Q21.33_DEL** | **-18q21.33** | 80 |
| **18Q21_NOT33** | **-18q21 & (# ^ -18q21.33)** | 49 |
| **VPS4B_LOSS** | **!vps4b \| -vps4b** | 99 |
| **IFNA1_MUT_ONLY** | **(# ^ -ifna1) & (!ifna1 \| /ifna1-)** | 9 |
| **MSI_FULL_DOWN** | **!msh2 \| -msh2 \| !msh6 \| -msh6 \| !mlh1 \| -mlh1 \| !pms2 \| -pms2 \| -pole \| !pole \| !pold1 \| -pold1 \| /pole:p286r,v411l,s459f \| <2mlh1** | 142 |
| **CHAN_MSI** | **@CHAN_MSI.txt **** | 99 |
| **KRAS_G12V** | **/kras:g12v** | 47 |
| **KRAS_G12D** | **/kras:g12d** | 72 |
| **KRAS_G12C** | **/kras:g12c** | 24 |
| **KRAS_G12ANY** | **/kras:g12?** | 173 |
| **KRAS_GOF** | **/kras+** | 223 |
| **ALK_GOF** | **/alk+** | 13 |
| **CTNNB1_GOF** | **/ctnnb1+** | 35 |
| **BRAF_GOF** | **/braf+** | 127 |
| **NRAS_12_13_61** | **/nras:g12?,g13?,q61?** | 91 |
| **HRAS_12_13_61** | **/hras:g12?,g13?,q61?** | 19 |
| **KRAS_12_13_61** | **/kras:g12?,g13?,q61?** | 206 |
| **PAN_RAS_12_13_61** | **/hras:?12? \| /kras:?12? \| /nras:?12? \| /hras:?13? \| /kras:?13? \| /nras:?13? \| /hras:?61? \| /kras:?61? \| /nras:?61?** | 315 |
| **APC_LOSS_TISSUE_AGE_REFINED** | **(!apc \| -apc) & ((!apc \| -apc) ^ (%lymph \| %uter \| ;ped))** | 93 |
| **APC_LOSS_BOWEL** | **(!apc \| -apc) & %bowel** | 62 |
| **APC_LOSS_BOWEL_KRAS_ACT** | **((!apc \| -apc) & %bowel) & /kras:a146?,a59t,g12?,g13?,k117n,q61?** | 36 |
| **APC_LOSS_BOWEL_KRAS_ACT_CDKN2A_NOTDEL** | **(!apc \| -apc) & %bowel & /kras:a146?,a59t,g12?,g13?,k117n,q61? & (=cdkn2a \| +cdkn2a)** | 29 |
| **CTNNB1_APC_LOSS_REF_5** | **(!apc \| -apc) & (# ^ ;Pediatric) & (# ^ %UTERUS) & (# ^ %LYMPHOID) & (# ^ -CDKN2A)** | 83 |
| **CTNNB1_APC_LOSS_REF_6** | **(!apc \| -apc) & (# ^ ;Pediatric) & (# ^ %UTERUS) & (# ^ %LYMPHOID) & %BOWEL** | 62 |
| **MSI_FULL_DOWN_BOWEL** | **(!msh2 \| -msh2 \| !msh6 \| -msh6 \| !mlh1 \| -mlh1 \| !pms2 \| -pms2 \| -pole \| !pole \| !pold1 \| -pold1 \| /pole:p286r,v411l,s459f \| <2mlh1) & %bowel** | 23 |
| **WRN_MSI_FULL_DOWN_REF_15** | **(!msh2 \| -msh2 \| !msh6 \| -msh6 \| !mlh1 \| -mlh1 \| !pms2 \| -pms2 \| -pole \| !pole \| !pold1 \| -pold1 \| /pole:p286r,v411l,s459f \| <2mlh1) & (# ^ %LYMPHOID) & !KMT2D & !TTN & (# ^ -9p21.3)** | 30 |

* *fully expanded definitions that do not rely on predefined profile names*

*** harvested from Supplementary Table 1 of Chan et al (2019)*

Locus scans for KRAS, RAB6A and ITGB1


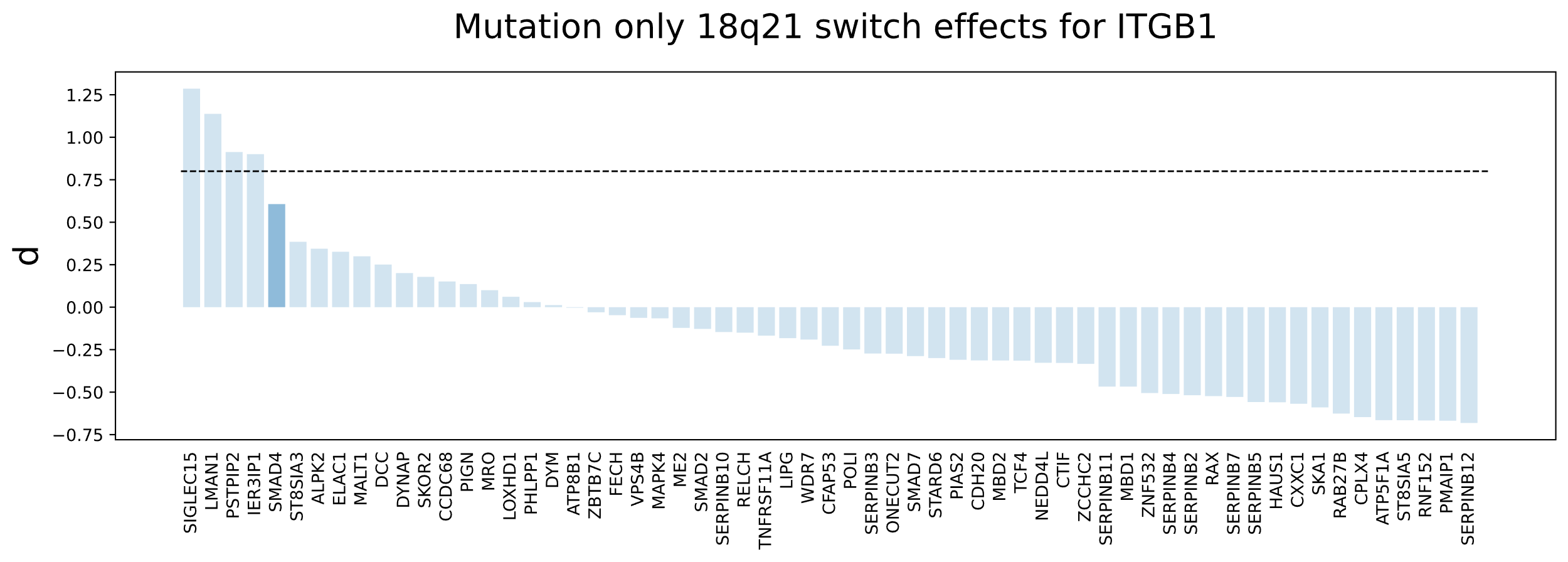

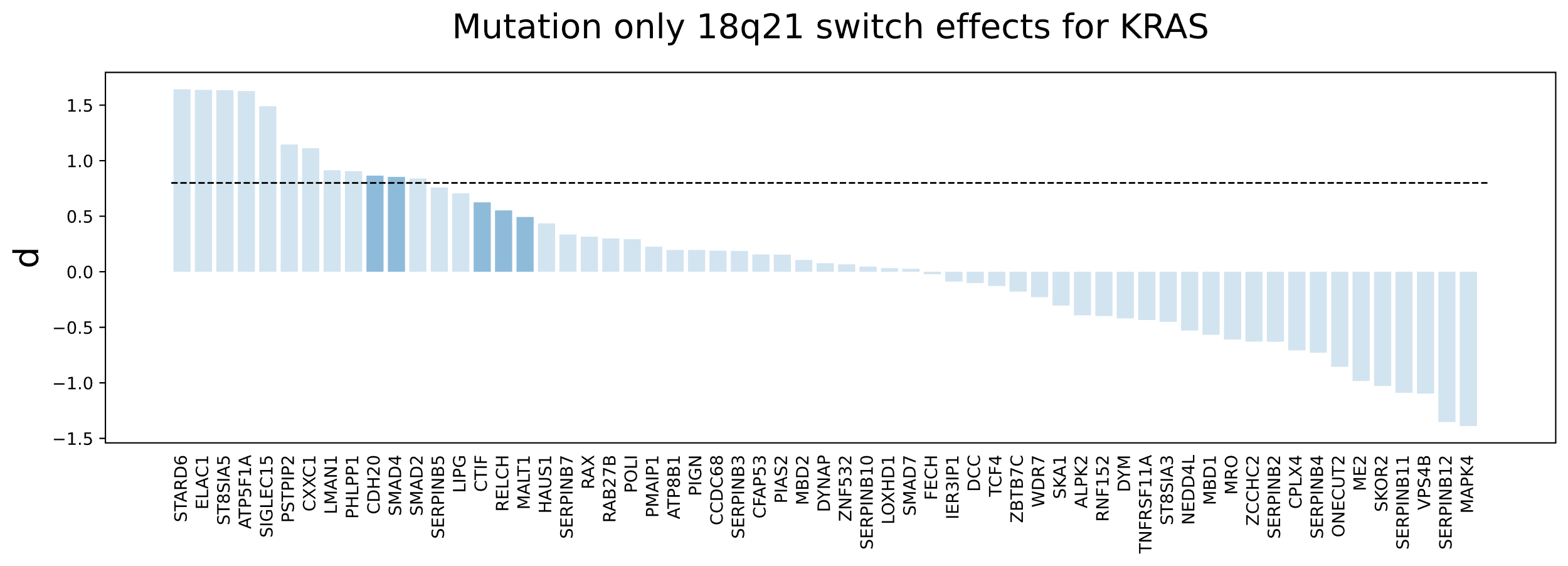

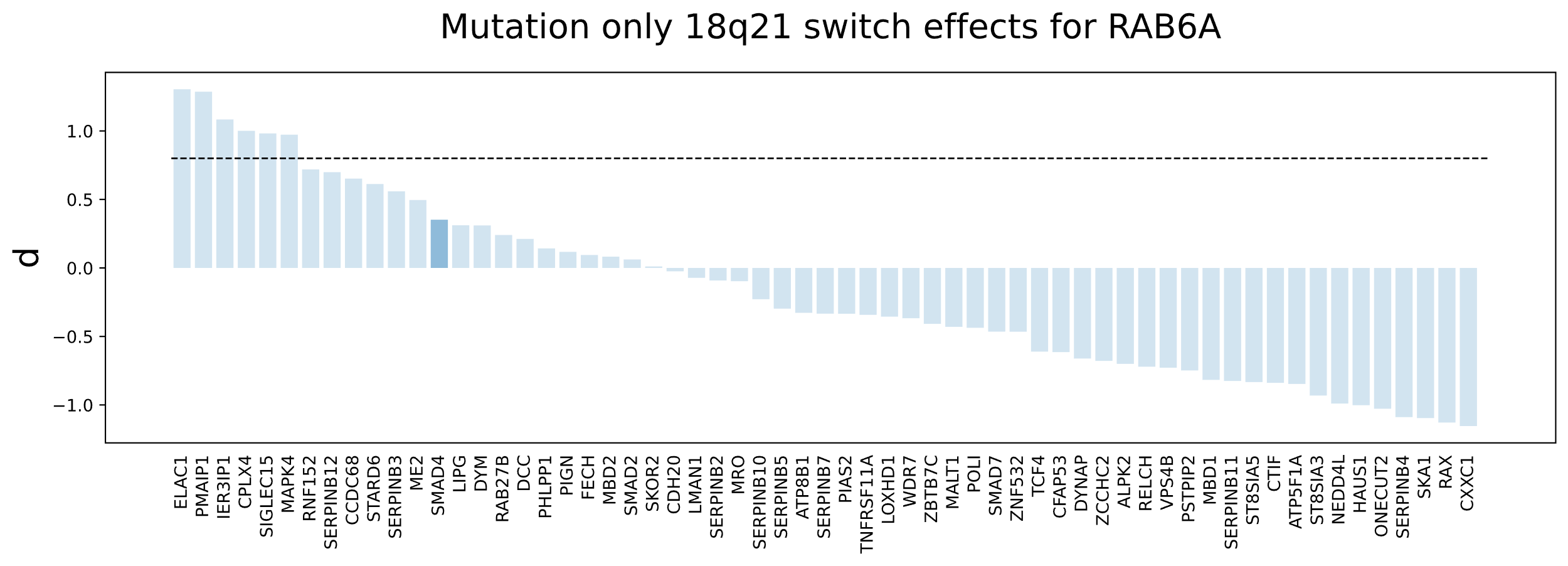


**Supplementary Databases**

**Supplementary Database 1.tsv** – List of 34256 GeneA:GeneB pairs where the |dep(GeneA)| < 0.65, and dep(GeneA) in cells with LoF/CNV-del of GeneB was significantly > than GeneB-WT cells (p < 0.05).

**Supplementary Database 2.tsv –** List of 2082 GeneA:GeneB pairs where GeneB has any literature annotated Gain-of-Function (GoF) mutation, and where dep(GeneA) in cells with GeneB-GoFwas significantly > than GeneB-WT cells ((p < 0.05 and d > 0.8).
